# Supplementary material for: Defining an Optimal Range of Centrifugation Parameters for Canine Semen Processing
Source: Animals (Basel). 2023 Apr 21;13(8):1421. doi: 10.3390/ani13081421 (PMC10135290; doi:10.3390/ani13081421)
Supplement: Supplementary file 1 [file animals-13-01421-s001.zip › animals-2299230-supplementary.pdf]

Supplementary Tables:

Table S1: Change in morphologic defects of spermatozoa (%) from baseline (T0 for initial raw semen evaluation) over time by centrifugation treatment groups. Treatment groups labelled as A: 400 g 5 min, B: 720 g 5 min, C: 900 g 5 min, D: 400 g 10 min, E: 720 g 10 min, F: 900 g 10 min. Time points T1: post-centrifugation, T2: 24 h of cooling, T3: 48 h of cooling. Different superscript letters (a, b, c) denote statistically significant ($p<0.05$) differences between time points for a given treatment group per sperm parameter. Black triangles ($^{\blacktriangle}$) or asterisks (*) denote statistically significant ($p<0.05$) difference between treatment groups within a given time point. Absence of markings within a row or column means no significant difference.

**Treatment groups**	**Percentage change from baseline for acrosome abnormalities (median ± SEM)**			**Percentage change from baseline for head abnormalities (median ± SEM)**			**Percentage change from baseline for midpiece abnormalities (median ± SEM)**			**Percentage change from baseline for tail abnormalities (median ± SEM)**		
	**T1**	**T2**	**T3**	**T1**	**T2**	**T3**	**T1**	**T2**	**T3**	**T1**	**T2**	**T3**
**A**	2.5±1.31^{a}	9.50±2.12^{b}	19.0±2.27^{c}	1.75±2.19	6.0±2.51	9.50±2.21	1.25±2.67^{a}	1.75±2.62^{a,b}	-3.25±1.73^{b}	-2.25±1.38	-1.0±1.14$^{\blacktriangle}$	-2.75±1.73
**B**	2.25±1.36^{a}	14.0±1.75^{b}	19.25±2.36^{c}	6.75±2.30^{a}	9.25±2.28^{a,b}	11.75±1.66^{b}	-2.50±2.51	-1.75±2.79	-3.25±1.62	-2.75±1.13	0±1.68	-1.25±1.51
**C**	3.75±1.42^{a}	14.0±1.80^{b}	17.75±2.51^{b}	4.75±2.18	9.25±2.36	6.25±2.32	0.25±0.96^{a,b}	-0.75±2.53^{a}	-4.50±1.56^{b}	-1.50±1.53	-4.0±0.62$^{\blacktriangle,*}$	-1.25±1.78
**D**	4.25±1.34^{a}	14.75±2.50^{b}	20.50±2.27^{b}	4.0±1.60^{a}	8.50±1.50^{a,b}	8.0±2.12^{b}	-3.0±2.43	-4.50±2.44	-2.75±1.74$^{\blacktriangle}$	1.0±1.29	0.75±1.55	-2.0±1.49
**E**	2.50±1.54^{a}	16.25±2.27^{b}	19.75±2.70^{b}	4.50±1.68	7.75±1.68	10.75±2.11	-3.75±1.98	-4.0±2.88	-3.50±2.15	3.25±0.95	-1.25±1.02*	-2.50±1.68
**F**	1.50±1.25^{a}	15.0±2.12^{b}	20.75±2.03^{b}	5.25±2.07	7.25±3.10	8.0±2.68	-3.0±2.52^{a,b}	-1.25±2.45^{a}	-6.0±1.38$^{b,\blacktriangle}$	-0.50±1.48	-3.0±1.34	-2.0±1.90
